# Supplementary material for: Eleven internal fixations for young vertical femoral neck fractures: A protocol for systematic review and network meta-analysis
Source: PLoS One. 2024 Sep 24;19(9):e0310971. doi: 10.1371/journal.pone.0310971 (PMC11421801; doi:10.1371/journal.pone.0310971)
Supplement: S2 File — (DOCX) [file pone.0310971.s002.docx]

**The search strategy for PubMed**

| #ID | Topic or intervention | Query |
| --- | --- | --- |
| #1 | Disease | ((("Femoral Neck Fractures"[Mesh]) OR (Femur Neck Fracture[Title])) OR (Femur Neck Fractures[Title])) OR (Femoral Neck Fracture[Title]) |
| #2 | Intervention | ((((((((((((Nailing, Intramedullary[Title/Abstract]) OR (Nailings, Intramedullary[Title/Abstract])) OR (Intramedullary Nailings[Title/Abstract])) OR (Intramedullary Nailing[Title/Abstract])) OR (Osteosynthesis, Fracture, Intramedullary[Title/Abstract])) OR (Intramedullary Fracture Fixations[Title/Abstract])) OR (Intramedullary Fracture Fixation[Title/Abstract])) OR (Fracture Fixations, Intramedullary[Title/Abstract])) OR (Fixations, Intramedullary Fracture[Title/Abstract])) OR (Fixation, Intramedullary Fracture[Title/Abstract])) OR ("Fracture Fixation, Intramedullary"[Mesh])) OR ("Internal Fixators"[MeSH Terms] OR "Fixator, Internal"[Title/Abstract] OR "Fixators, Internal"[Title/Abstract] OR "Internal Fixator"[Title/Abstract] OR "Fixation Devices, Internal"[Title/Abstract] OR "Device, Internal Fixation" [Title/Abstract] OR "Devices, Internal Fixation" [Title/Abstract] OR "Internal Fixation Device" [Title/Abstract] OR "Internal Fixation Devices")) OR ((((((((((Fracture Osteosyntheses[Title/Abstract]) OR (Osteosyntheses, Fracture[Title/Abstract])) OR (Fracture Osteosynthesis[Title/Abstract])) OR (Osteosynthesis, Fracture[Title/Abstract])) OR (Internal Fracture Fixations[Title/Abstract])) OR (Internal Fracture Fixation[Title/Abstract])) OR (Fracture Fixations, Internal[Title/Abstract])) OR (Fixations, Internal Fracture[Title/Abstract])) OR (Fixation, Internal Fracture[Title/Abstract])) OR ("Fracture Fixation, Internal"[Mesh])) |
| #3 | Study design | "randomized controlled trial"[pt] OR "controlled clinical trial"[pt] OR randomized[tiab] OR placebo[tiab] OR "drug therapy"[sh] OR randomly[tiab] OR trial[tiab] OR groups[tiab] |
| #4 | Final query | #1 AND #2 AND #3 |
